# Dissecting Gut‐Microbial Community Interactions using a Gut Microbiome‐on‐a‐Chip

**DOI:** 10.1002/advs.202302113

**Published:** 2024-02-27

**Authors:** Jeeyeon Lee, Nishanth Menon, Chwee Teck Lim

**Affiliations:** ^1^ Institute for Health Innovation and Technology (iHealthtech) National University of Singapore Singapore 117599 Singapore; ^2^ Department of Biomedical Engineering National University of Singapore Singapore 117583 Singapore; ^3^ Mechanobiology Institute National University of Singapore Singapore 117411 Singapore

**Keywords:** gut microbiota, gut‐microbial community interactions, gut‐on‐a‐chip, inter‐microbial interactions, organ‐on‐a‐chip, Microbiome

## Abstract

While the human gut microbiota has a significant impact on gut health and disease, understanding of the roles of gut microbes, interactions, and collective impact of gut microbes on various aspects of human gut health is limited by the lack of suitable in vitro model system that can accurately replicate gut‐like environment and enable the close visualization on causal and mechanistic relationships between microbial constitutents and the gut. , In this study, we present a scalable Gut Microbiome‐on‐a‐Chip (GMoC) with great imaging capability and scalability, providing a physiologically relevant dynamic gut‐microbes interfaces. This chip features a reproducible 3D stratified gut epithelium derived from Caco‐2 cells (µGut), mimicking key intestinal architecture, functions, and cellular complexity, providing a physiolocially relevant gut environment for microbes residing in the gut. Incorporating tumorigenic bacteria, enterotoxigenic *Bacteroides fragilis* (ETBF), into the GMoC enable the observation of pathogenic behaviors of ETBF, leading to µGut disruption and pro‐tumorigenic signaling activations. Pre‐treating the µGut with a beneficial gut microbe Lactobacillus spp., effectively prevent ETBF‐mediated gut pathogenesis, preserving the healthy state of the µGut through competition‐mediated colonization resistance. The GMoC holds potential as a valuable tool for exploring unknown roles of gut microbes in microbe‐induced pathogenesis and microbe‐based therapeutic development.

## Graphical abstract

A scalable Gut Microbiome‐on‐a‐Chip (GMoC) that faithfully recreates a physiologically relevant dynamic gut‐microbes interface is presented. The GMoC provide a biomimetic 3D gut model (μGut), closely mimicking key features of the intesine and enabling the visualization of gut microbial behaviors, interactions, and their collective influence on gut health and disease through high‐magnification imaging. The GMoC can offer valuable insights into dissecting microbe‐induced pathogenesis and developing microbe‐based therapeutics to enhance overall gut health.

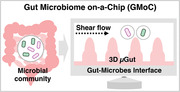

## 1 Introduction

The human gastrointestinal (GI) tract is home to a diverse population of microbes that play critical roles in human health and disease.^[^ ^1^ ^]^ Recent evidences have drawn significant attention to the impact of gut microbiota on various health conditions, including inflammatory bowel disease (IBD),^[^ ^2^ ^]^ metabolic diseases,^[^ ^3^ ^]^ neurological disorders,^[^ ^4^ ^]^ and cancer.^[^ ^5^ ^]^ Despite the growing interest in the gut microbiome, many questions about the roles and interactions of gut microbes, mechanisms of their resilience or resistance to environmental factors, and their impact on human health and disease, remain unanswered. While a deeper understanding of the gut microbiota holds immense potential to answer these questions, research progress in this field is delayed due to the limited understanding of gut microbe's causal and mechanistic interactions with their other surrounding microbes and the gut.

In order to gain a comprehensive understanding of the intricate relationships between gut microbial constituents and their host, it is crucial to have a model system that faithfully recapitulates the key structures and functions of the human intestine, allowing for the colonization of gut microbial inhabitants. The tools need to provide insights into the growth patterns of gut microbes, the development of distinctive behaviors with both pathogenic and beneficial potential, the intricate interactions among various microbial species leading to the formation of specific microbial communities (groups of microorganisms) and the overall impact of these microbial interactions on gut health and disease at the mechanistic level. By enhancing the visualization of the dynamic interface of gut‐microbial communities, we can effectively distinguish between different microbial species and visualize the spatial organization and dynamics of these communities in response to stimuli.

Visualization offers a deeper and more comprehensive understanding of the collaborative and competitive interactions that occur between gut microbial species, forming ecologically distinct entities and leading to diverse outcomes in gut health. This understanding can pave the way for new therapeutics possibilities, not only in uncovering new targets within the mechanisms of microbe‐induced gut disease but also in potentially facilitating microbe‐induced therapeutics such as the development of effective microbial communities for modulating the gut microbiota as novel therapeutic treatments.

Although in vivo animal models offer numerous advantages for studying gut microbiota, they face limitations in dissecting and visualizing causal and mechanistic relationships. This is primarily due to the complex interactions among epithelial and neighboring cells, as well as the presence of resident and transient intestinal microbes.^[^ ^6^ ^]^ While in vitro modeling of the gut‐microbe interface may not quite fully replicate the cellular complexity of the human intestine or the diverse gut microbiota, it can still serve as a suitable model for dissecting intricate causal and mechanistic relationships between microbial species and the gut. However, recreating an in vitro gut‐microbe model necessitates careful consideration of several crucial factors.

Host components, such as a 3D structured habitat, exert a significant influence on microbial growth, behavior, and interactions with host cells within a spatial context. Evidence has demonstrated differences in microbial behavior between 2D planar surfaces to 3D structures,^[^ ^7^ ^]^ underscoring the importance of host architecture in 3D in vitro modeling. The 3D structure of the gut plays a crucial role in establishing microbial communities composed of different microbial species, as the spatial organization of these communities within a 3D substrate influences their functions and has a distinctive impact on the gut under various stimuli.^[^ ^8^ ^]^ Mucin production by the gut serves as a critical primary barrier against microbial invasions in the intestinal epithelium,^[^ ^9^ ^]^ while also functioning as an attachment site^[^ ^10^ ^]^ and a source of carbon^[^ ^11^ ^]^ for enteric bacteria. Mechanical forces, such as shear flow and peristaltic motion, which mimic intraluminal movement, play a vital role not only in facilitating the co‐culturing of gut cells with bacteria but also in determining the behaviors of gut microbes at individual and multicellular levels.^[^ ^12^ ^]^ Furthermore, a scalable and reproducible gut model system enables the simultaneous testing of a large number of diverse microbes with different properties, ensuring minimal variability and enhancing the efficiency and accuracy of data collection.

While several in vitro gut model systems have been developed, they do not fully meet all the important criteria to create a gut‐microbial interface. For example, bioreactor‐based models^[^ ^13^ ^]^ are useful for investigating the composition and functions of gut microbiota, but their inability to incorporate host components limits the study of their effect on host cells. Precision‐cut tissue slides (PCTs)^[^ ^14^ ^]^ provide a tissue model with cellular complexity close to the human intestine, but their reproducibility and scalability are hampered by issues such as tissue procurement, viability and donor‐to‐donor variability. Intestinal organoids^[^ ^15^ ^]^ can establish 3D structured intestines with cellular complexity and the ability to support microbial culture. However their low‐throughput and lack of mechanically relevant stimuli limits their application.^[^ ^16^ ^]^ Moreover, none of these models provide an easy way to visualize the interaction between the gut and microbial constituents.

The gut‐on‐a‐chip technology offers powerful tools to mimic important features of gut physiology^[^ ^17^ ^]^ necessary to create in vitro gut‐microbe interface for investigating both indirect^[^ ^18^ ^]^ or direct^[^ ^19^ ^]^ interactions. For example, the two‐chamber design of the gut chip,^[^ ^18a,b^ ^]^ where gut cells and microbial species are cultured in separate membranes, can provide conditions resembling the gastrointestinal human‐microbe interface, including anaerobic oxygen controls. However, the absence of direct interactions between the gut and microbes are only capable of providing gut responses through secreted metabolites of bacteria, hindering the acquisition of information stimulated by direct contact. Additionally, the 3D spatial organization of the gut microbial community can not be recapitulated in indirect culture methods, despite its importance in determining the functions of the gut microbiota. On the other hand, the previously developed gut chip model with a stacked channels configuration^[^ ^19^ ^]^ has the advantage of directly co‐culturing gut cells and microbes under mechanical stimuli, alongside other relevant organs.^[^ ^19a–g^ ^]^ It serves as a useful tool to investigate gut bacteria‐induced gut response as well as their impact on other organ systems.

However, the juxtaposition of the top and bottom cell culture channels poses challenges for in situ high‐magnification imaging to visualize microbial constitutents or real‐time imaging of the gut‐microbe interface, necessitating another dismantling or slicing process of the device. Unfortunately, these steps disrupt the bacteria or bacterial community colonized within the 3D gut space, resulting in the loss of information regarding spatial organizations or interactions between gut microbial species. In addition to these limitations, the design complexity of these models limits scalability or parallelization for high throughput applications.

Here, we present a novel Gut Microbiome‐on‐a‐Chip (GMoC) that offers high resolution imaging capabilities, enabling the visualization of microbial growth, their unique behaviors, interactions within the microbial community, and their individual as well as collective impact on a gut. Our GMoC incorporates a scalable and reproducible design,  encompassing essential features forrecapitulating gut‐microbial interfaces. These features include shear flow and a biomimetic 3D structured gut that mimic key attributes of the human intestine, creating an environment suitable for gut microbial inhabitants. The incorporation of high‐magnification imaging in our GMoC enables us to observe the detrimental or protective behaviors and mechanisms of gut microbes and the community on the Caco‐2 derived 3D stratified gut epithelium (μGut). Our study highlights the potential of the GMoC in uncovering the unknown roles of individual gut microbe, as well as the collective behaviors of gut microbial consortia that significantly impact gut health and disease.

## 2 Results

### 2.1 Design of the Gut Microbiome‐on‐a‐Chip (GMoC) with Shear‐Induced 3D Structured µGut with Key Attributes of the Physiological Intestinal Epithelium

**Figure 1:**
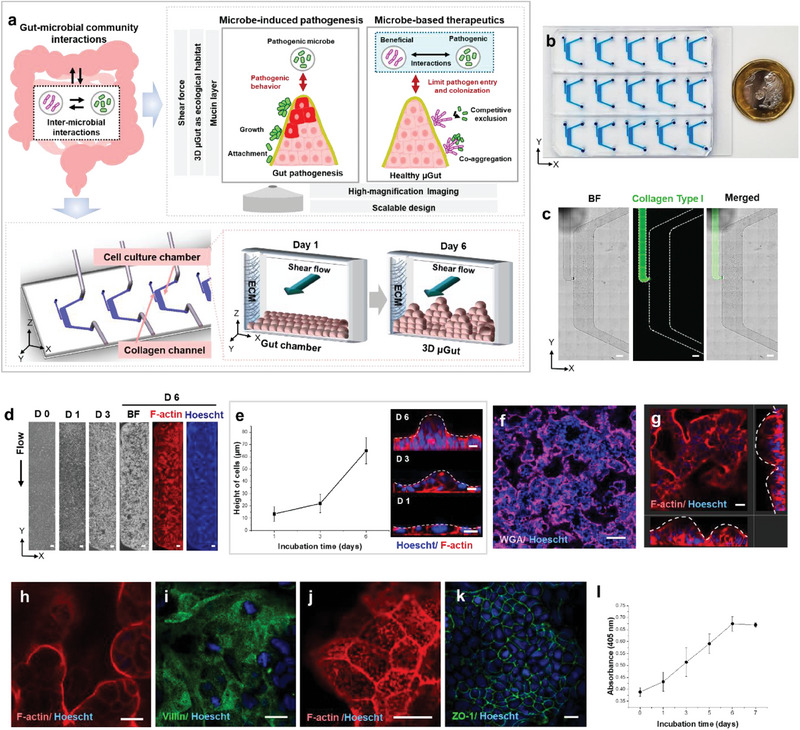
The GMoC showcasing a dynamic 3D µGut‐microbes interface, where the shear‐induced 3D µGut effectively mimics the key structures and functions of the human intestine. a) The GMoC consisting of µGut chamber consisting of the cell culture chamber and the collagen channel, features a dynamic gut‐microbiome interface. The chip provides shear flow and the ECM‐supported self‐structured 3D µGut capable of producing mucin, supporting microbial attachment and growth over an extended period. The chip offers compatibility with high‐magnification imaging to visualize a single microbe, distinguish different bacterial species and their inter‐microbial interaction as well as the influence of a microbial community on the µGut. Our GMoC allows for dissecting causal and mechanistic roles of gut microbes for microbe‐induced pathogenesis (Figures 3 and 4) and microbe‐based therapeutics (Figures 5 and 6). b) The GMoC featuring high scalability and integrability to high‐magnification imaging. c) Brightfield and fluorescence images of the chip showcasing the collagen (FITC) channel and the culture chamber (Scale bar, 500 µm). d) Time‐lapse images of the Caco‐2 derived µGut formation in the GMoC over 6 days. Fluorescence images of the µGut on Day 6 show uniform cell density across the length of the chip (Scale bar, 200 µm). e) Cell height measurement and cross‐section images of the µGut over 6 days showing morphogenesis of a villus‐like structure. f) Confocal immunofluorescence top‐view image of the 3D stratified µGut epithelium (Scale bar, 100 µm) consisting of g) continuous crypt‐villus units. h) Top view of villi‐like structures, and i) brush border covered with j) microvilli in the µGut. k) Expression of tight junction (ZO‐1) indicating gut barrier formation. l) Steady increase of aminopeptidase activity of the μGut during the self‐morphing process. All scale bars represent 20 µm unless otherwise indicated.

Our goal is to establish a dynamic gut‐microbe interface to closely monitor the attachment, growth, and pathogenic or beneficial behaviors of gut microbes and the microbial communities on the gut epithelium, as well as their influence on the gut from a morphological to a mechanistic level (**Figure** **1**a). Key considerations for the design included ensuring ease of fabrication to facilitate device parallelization, the ability to generate a consistent microenvironment for a physiologically relevant 3D gut epithelium, and easy integration with high‐resolution and high‐magnification microscopy for in vitro visualization. This visualization capability extends to in‐situ monitoring of individual bacteria and multicellular assemblies, as well as distinguishing different microbial species within the microbial communities, all without the need to dismantle the experimental set‐up and slice the gut sample. Shear flow not only provides the mechanical stimuli required for self‐structuring of the 3D µGut from the Caco‐2 cell monolayer, but also simulates the intraluminal flow experienced by the gut microbes.

The GMoC developed here comprises a polydimethylsiloxane (PDMS) chip with multiple rows of laterally arranged µGut culture chambers (Figure 1b) bonded to a standard glass coverslip ensuring easy integration with high‐magnification microscopy. Furthermore, the gas permeability of PDMS allows for flexible and effective control over the oxygen‐controlled microenvironmental conditions for different types of gut microbes requiring varying levels of oxygen. Each µGut chamber consists of a micro‐channel to culture the 3D µGut interconnected to an adjacent collagen channel to house a 3D collagen gel matrix by a 150 µm wide opening designed to confine the collagen gel within the respective channel. Our experimental observations indicate that while collagen does not diffuse into the µGut chamber, the presence of collagen gel facilitates the attachment, proliferation , and long‐term culture of the µGut cells^[^ ^20^ ^]^ (Figure 1a,c). Physiologically relevant shear stress (0.034 dyne cm^−2^; reported in vivo shear stress (≈0.002–0.08 dyne cm^−2^)^[^ ^21^ ^]^ was generated within the culture chamber to recreate the dynamic environment of the human intestine and prevent bacterial overgrowth (Figure S1 and Video S1, Supporting Information).

The µGut was generated from the Caco‐2 cell line under perfusion and a time‐dependent increase in cell density was observed over 6 days. Also, the Caco‐2 cells self‐morphed into a multi‐layered gut epithelium throughout the length of the chip in contrast to cell monolayer in static culture (Figure 1d ; Figure S2, Supporting Information). The morphogenesis of villi‐like structure and formation of the distinct crypt‐villus axis was visualized through time‐lapse confocal microscopy. Shear stress initially induced polarization of the Caco‐2 cell monolayer within 24 h, which manifested as increased cell height (≈14 µm), and formed 3D stratified epithelium (Figure 1d,e). Upon closer observation, crypt‐villus units with basal crypt (Figure 1f,g) and protruding finger‐like structures (height ≈60–70 µm) (Figure 1h and Video S2, Supporting Information) were noticed throughout the cell culture channel within 6–7 days, thereby forming the µGut epithelium. This is in sharp contrast to the cell monolayer observed in Transwell™ or static culture systems (Figure S2, Supporting Information). This contradicts several gut‐on‐chip models that emphasized the importance of a fluid flow along the basal surface of the cell for the formation of 3D villi‐like cell organization^[^ ^22^ ^]^ In this study, we noted that shear stress along the apical surface of the Caco‐2 cells can initiate polarization and cell proliferation into villi‐like organization. These results corroborate with recent observations where Caco‐2 cell polarization and 3D villi‐like morphogenesis were reported upon activation through apical shear stress.^[^ ^23^ ^]^ Apart from morphological features, the µGut displayed key attributes of a physiological intestinal epithelium including the presence of a well‐differentiated brush border (Figure 1i) covered with microvilli (Figure 1j), and well‐defined tight junction indicated by the immunofluorescence staining of Zonula Occludens‐1 (ZO‐1) (Figure 1k). Also, a steady increase of the brush border aminopeptidase activity was observed across the course of the experiments suggesting that the µGut epithelium closely recapitulated the digestive function of the human intestine (Figure 1l ). Taken together, we developed the GMoC to create multiple copies of the morphologically and functionally relevant human 3D gut model. The architecture of the chip enables easy expansion to a high‐throughput culture platform, with a 96‐well or a 384‐well plate format, to facilitate large‐scale, simultaneous testing of each and combinations of the microbial community.

### 2.2 Effects of Dynamic Environment on µGut Differentiation, Spatial Patterning and Mucin Production

The intestinal epithelium is a continuously self‐renewing and differentiating tissue with specialized cells necessary to perform key intestinal functions.^[^ ^9^, ^24^ ^]^ The stem cells from the crypt differentiate into functionally specialized cells and gradually migrate up toward the villus tip.^[^ ^24^, ^25^ ^]^ Differentiated cells such as enterocytes, goblet cells, and enteroendocrine cells are spatially arranged in the villus compartment, and proliferating cells such as stem cells and differentiated secretory paneth cells are organized in the crypt. Rapidly proliferating, transit‐amplifying (TA) cells are located in the upper region of a crypt.^[^ ^24^, ^25^ ^]^ The spatially organized, specialized gut epithelial cells play critical roles in forming the gut barrier, segregating the gut microbiota in the lumen,^[^ ^9^ ^]^ while also generating a dynamic microenvironment that provides a distinct ecological niche for different types of gut microbial species.^[^ ^26^ ^]^

**Figure 2:**
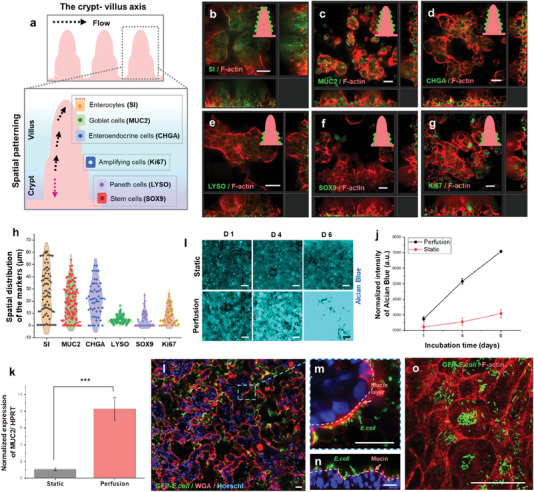
The shear‐induced differentiation, spatial patterning, and mucin secretion in the µGut facilitate the culture of gut microbes by establishing an environment conducive to their growth. a) A schematic illustrating the crypt‐villus architecture of the human intestine and the localization of specialized cells. Immunofluorescence confocal imaging showed that the µGut contains various major types of differentiated cells such as b) enterocytes (sucrase‐isomaltase (SI)), c) goblet cells (MUC2), d) enteroendocrine cells (chromogranin A (CHGA)), e) paneth cells (Lysozyme (LYSO)), f) stem cells (SOX9), g) proliferating cells mimicking transit‐amplifying (TA) cells (Ki67). The inset figures represent the location of the gut markers (green) in the crypt‐villus axis of the µGut (pink). h) Spatial distribution of the six major intestinal cell types in the crypt‐villus axis. i) Time‐dependent mucin secretion of the µGut stained with Alcian blue. j) Intensity of Alcian blue stain of the µGut culture under the static and perfused condition. k) Relative MUC2 gene expression between the static and perfusion µGuts (***p < 0.001). l) A large image of the GFP*‐E. coli* colonized µGut after 72 h incubation (Scale bar, 100 µm) showcasing attachment of GFP*‐E. coli* on m, n) the mucin layer, and o) µGut brush border. All scale bars represent 30 µm unless otherwise indicated.

In our Caco‐2 derived µGut, an abundance of differentiated intestinal epithelial cell types including enterocytes (sucrase‐isomaltase; SI),^[^ ^27^ ^]^ goblet cells (Mucin 2; MUC2),^[^ ^28^ ^]^ enteroendocrine cells (Chromogranin A; CHGA),^[^ ^29^ ^]^ paneth cells (Lysozyme; LYSO),^[^ ^30^ ^]^ stem cell (SOX9)^[^ ^31^ ^]^ and proliferating cells (Ki67)^[^ ^32^ ^]^ were observed under a perfused state, indicating shear‐induced differentiation (**Figure** **2**a). These results are in alignment with previous studies demonstrating the differentiation of Caco‐2 cells into major types of intestinal cells under controlled culture conditions or stimuli.^[^ ^22^, ^23^, ^33^ ^]^ The application of shear flows further promoted the spatial patterning of the differentiated cells in the µGut crypt‐villus axis where enterocytes (SI), goblet cells (MUC2), and enteroendocrine cells (CHGA) were distributed along the height of the villus (Figure 2b–d) while both paneth cells (LYSO) and stem cells (SOX9) were predominantly spotted near the base, similar to their locations in vivo (Figure 2e,f). On the contrary, static culture yielded lower numbers of these differentiated cells in a 2D organization (Figure S3, Supporting Information). Furthermore, we noticed proliferative cells (Ki67+) located at the mid‐section of the crypt‐villus axis, mimicking the location of the rapidly proliferating TA cells (Figure 2g). We further extracted the locations of the six differentiated cell types from multiple images and mapped their positions along the height of the villi. Differentiated cells, such as enterocytes, goblet cells, and enteroendocrine cells were located within the range of 0–70 µm along the height of the villi. In contrast, stem cells, paneth cells, and amplifying cells were preferentially distributed within the range of 0–25 µm in height. This highlights the perfusion‐induced spatial patterning of the major six types of differentiated gut cells along the crypt‐villus axis in the μGut (Figure 2h).

Intestinal goblet cells produce mucin, serving as the primary line of defense against microbial invasions,^[^ ^9^ ^]^ while also acting as attachment sites^[^ ^10^ ^]^ and a source of carbon for gut microbes.^[^ ^11^ ^]^ The alcian blue staining of the µGut epithelium exhibited a shear‐induced mucin production, resulting in ≈ a 2.5‐fold increase by Day 6 compared to the static culture condition. This finding aligns with a previous study demonstrating mucin secretion by Caco‐2 cells in response to mechanical stimulation^[^ ^22a^ ^]^ (Figure 2i,j).

Despite Caco‐2 being reported as a MUC2‐deficient cell line,^[^ ^34^ ^]^ our results revealed that MUC2 gene expression in the perfused µGut was ≈8‐fold higher than in static culture, suggesting the impact of mechanical stimuli on altering the gene expression of the Caco‐2 cells (Figure 2k). The increased mucin production in the µGut promotes GFP‐*E. coli* colonization and growth by forming microcolonies on the mucin layer without invading µGut cells for up to 72 h (Figure 2l–o). These results highlight the µGut's self‐producing capability of mucin, contributing to establishing the gut‐bacteria interface and enabling extended co‐culturing, which was unachievable with static gut models. Overall, our results demonstrate that mechanical stimuli provided by our GMoC promote the differentiation and spatial patterning of the µGut, successfully recreating the biomimetic 3D gut epithelium.

### 2.3 Colonization of Tumorigenic ETBF in µGut Develop Pathogenic Behavior and µGut Disruption

**Figure 3:**
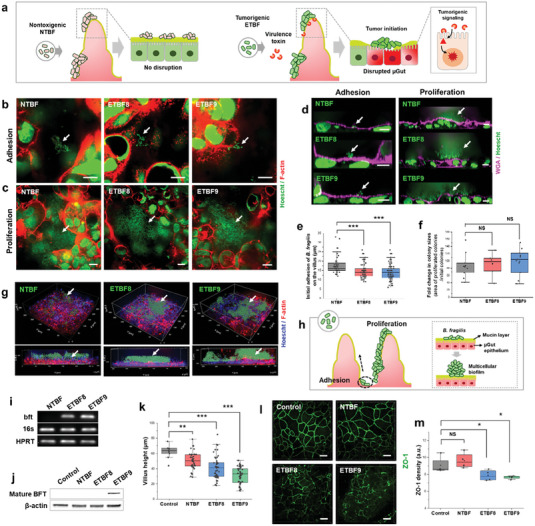
The adhesion, growth, and development of pathogenic behavior of ETBF lead to disruptions in the 3D µGut. a) Schematics illustrating the varying degrees of µGut disruption induced by different strains of *B. fragilis*. b) Adhesion (3 h) and c) proliferation (24 h) of *B. fragilis* strains on the µGut surface. d) Immunofluorescence cross‐section images of the µGut‐ *B. fragilis* interface showing bacterial adhesion and proliferation on the mucin layer. e) Spatial distribution of *B. fragilis* strains along the crypt‐villus axis in the 3D µGut. f) Fold changes in colony sizes between adhesion and proliferation stages for various *B. fragilis*. g) 3D reconstruction, and h) an illustration of the µGut‐ *B. fragilis* interface, showcasing a multicellular bacterial biofilm formed on the   µGut. i) Agarose gel electrophoresis of the bft PCR products, and j) Western blotting of mature BFT toxin (≈20 kDa) extracted from µGuts colonized by NTBF, ETBF8 and ETBF9. k) Changes in villi heights of the µGut colonized with various *B. fragilis* strains. l) ETBF induced ZO‐1 Redistribution, and m) its calculated density in the µGuts. P‐value indicate *p < 0.05; **p < 0.01; ***p < 0.001, NS→ *p* > 0.05. All scale bars represent 10 µm unless otherwise indicated.

*Bacteroides fragilis* is anaerobic bacteria that is commonly found in the colon, exhibiting strain diversity and varying levels of pathogenic potential in the human intestine.^[^ ^35^ ^]^ Enterotoxigenic strains of the bacteria (ETBF) produce BFT toxin that causes mucosal disruption, leading to the development of acute diarrheal and inflammatory bowel disease, as well as pre‐oncogenic signaling events that can contribute to colorectal cancer. On the other hand, nontoxigenic strains of *B. fragilis* (NTBF) are symbiotics and assist in maintaining intestinal homeostasis^[^ ^36^ ^]^ (**Figure** **3**a). To date, no in vitro co‐culture models have demonstrated the ability to recapitulate the live ETBF‐gut interface and visualize their pathogenic behaviors on the gut.

To demonstrate the capability of our GMoC in recapitulating and visualizing microbe‐induced gut pathogenesis, we conducted a proof‐of‐principle study using *B. fragilis* as our model pathogen. Two enterotoxigenic B. fragilis (ETBF) strains,

ATCC 43858 (ETBF8) and ATCC 43859 (ETBF9), along with a non‐BFT producing control NTBF, ATCC 25285 (NTBF), were selected to study the strain‐specific pathogenic behavior of *B. fragilis*. Optimization of culture conditions was a prerequisite for establishing µGut‐*B. fragilis* interface due to distinct oxygen requirements between anaerobic *B. fragilis* and µGut requiring normoxic condition (20% O_2_). Considering oxygen gradients ≈3–11 mm Hg (0.4%–2%) in the lumen of ascending and sigmoid colon^[^ ^37^ ^]^ and the characteristics of *B. fragilis* being aerotolerant^[^ ^38^ ^]^ up to 2% oxygen concentration,^[^ ^39^ ^]^ we examined 1% oxygen concentration as the culture condition to co‐incubate *B. fragilis* and the established µGut grown under normoxia for 6 days in the GMoC. A minimal effect on *B. fragilis* viability and µGut epithelial morphology (villi height) was observed after 24 h of hypoxic incubation (Figures S4 and S5, Supporting Information). Nonetheless, a two‐fold increase in HIF‐1α expression in the µGut cells was noted, indicating the presence of hypoxic stress (p < 0.001) (Figure S6, Supporting Information). After considering that a higher level of oxygen (> 2%) can inhibit the growth of *B. fragilis* ^[^ ^39^ ^]^ and that oxygen concentration below 1% or an incubation time exceeding 24 h, could adversely affect µGut integrity, we determined that optimal condition for co‐culturing µGut‐*B. fragilis* is 24 h at 1% hypoxia with media perfusion.

Initially, attachment of NTBF, ETBF8, and ETBF9 to the µGut was observed as small bacterial clusters after 3 h, which grew into larger clusters within the µGut after 24 h, indicating the growth of all bacterial strains (Figure 3b,c, and Videos S3–S5, Supporting Information). Cross‐sectional images showed the initial attachment of *B. fragilis* to the mucin layer of the µGut, followed by the formation of multicellular biofilms after 24 h (Figure 3d and Video S6, Supporting Information), consistent with biofilms previously observed in inflammatory bowel disease (IBD) patients^[^ ^40^ ^]^ and other animal studies.^[^ ^41^ ^]^ The initial binding of *B. fragilis* was found to be spatially localized near the crypt of the µGut (12–17 µm in height) (Figure 3e). ETBF exhibited a slightly lower positioning compared to NTBF, possibly due to mucus degrading activity of ETBF strains, resulting in a thinner mucin layer in ETBF colonized µGut.^[^ ^41b^ ^]^ Analysis of bacterial colony size between the adhesion and proliferation stages showed an 85–100 folds increase, indicating a similar rate of bacterial proliferation across all three strains (Figure 3f). The 3D visualization of the µGut‐*B. fragilis* interface revealed the sporadic distribution of thick bacteria biofilm formation throughout the µGut for all strains (Figure 3g). These observations suggest that initial *B. fragilis* attachment occurred in the crypt region, followed by active proliferation along the height of the villi, resulting in a denser biofilm that covered the µGut surface (Figure 3h).

ETBFs strains are known to carry the bft virulence gene,^[^ ^36a^ ^]^ which encodes the metalloprotease protein toxin known as BFT.^[^ ^36^, ^42^ ^]^ Upon translation, this BFT toxin is responsible for inducing morphological damage and disrupting the gut barrier by binding to intestinal epithelial receptors.^[^ ^43^ ^]^ The virulent bft gene was previously identified in a mucosal biofilm of familial adenomatous polyposis harboring ETBF^[^ ^41b^ ^]^ and ETBF‐infected mice models.^[^ ^41^, ^44^ ^]^ We investigated the presence of the bft gene in the µGuts colonized with ETBFs and NTBF strains through PCR amplification and subsequent agarose gel electrophoresis. ETBF8 and ETBF9 but not NTBF colonized µGuts contained the virulent bft gene (Figure 3i), despite presence of bacteria in all three models, as indicated by the bacteria‐specific 16s rRNA gene. The ≈20 kDa active BFT toxin which is cleaved from inactive ≈44 kDa pre‐BFT,^[^ ^36^, ^42^ ^]^ was exclusively present in the µGut colonized with ETBF9 but not with NTBF and ETBF8 (Figure 3j). Despite both ETBF8 and ETBF9 exhibiting the presence of the bft gene (Figure 3i) and evidence of BFT production by ETBF8,^[^ ^42^, ^45^ ^]^ the considerable difference in mature BFT levels observed may be attributed to variations in BFT expression between ETBF strains under different culture conditions.^[^ ^46^ ^]^ BFT toxin binds to an intestinal epithelial receptor and altered cell morphology and induces ZO‐1 redistribution^[^ ^43^ ^]^ (Table S1, Supporting Information). ETBF8 and ETBF9 colonization in µGut resulted in stunted villi heights of ≈41 and ≈31 µm, respectively with NTBF colonization inducing a marginal decrease in villus height (≈50 µm) compared to the bacteria‐free control (≈62 µm) (Figure 3k). Additionally, redistribution of the tight junction protein (ZO‐1), was observed in ETBF8 and ETBF9 colonized µGuts, while bacteria‐free control and NTBF colonized µGut remained unaffected (Figure 3l,m; Figure S8, Supporting Information). These results agree with previous studies that have reported morphological damages in intestinal cells and ZO‐1 redistribution during the early stages of ETBF‐induced gut pathogenesis, using BFT‐treated cells^[^ ^47^ ^]^ and in vivo models.^[^ ^48^ ^]^ No µGut disruption was observed due to the hypoxic environment (Figures S6 and S7, Supporting Information). Overall, despite similar bacterial density and colonization behavior of NTBF and ETBFs in the µGut, ETBF colonization induced detrimental disruption to the 3D architecture and barrier integrity of µGut. Specifically, ETBF9, induced severe morphological damages, indicating a strong correlation between the amount of BFT and ETBF‐induced µGut pathogenesis.

### 2.4 Tumorigenic ETBF Initiates Multiple Colorectal Tumor Signaling Pathways in µGut

Colonization of tumorigenic ETBF to the intestinal epithelium can trigger multiple signaling pathways, including E‐cadherin cleavage,^[^ ^49^ ^]^ Wnt/β‐catenin pathway,^[^ ^50^ ^]^ pStat3 signaling,^[^ ^51^ ^]^ and NF‐κB signaling,^[^ ^52^ ^]^ which have been linked to pre‐oncogenic events for colorectal cancer (CRC).^[^ ^36^, ^53^ ^]^ While these events have not been induced by live ETBF in in vitro gut models, we investigated whether the CRC signaling events reported in ETBF‐colonized in vivo models and BFT‐treated cells could be replicated in our µGut model. To exclude the effects of hypoxia on the target pathways , we assessed the activation of all key pathways described in this study under both normoxic and hypoxic conditions (Figure S8, Supporting Information).

**Figure 4:**
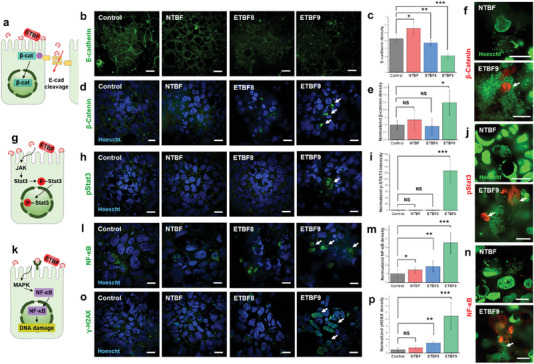
Activation of multiple tumorigenic signaling pathways induced by ETBF colonization in the µGut. a) A schematic of the BFT‐induced E‐cadherin cleavage and β‐catenin nuclear translocation triggered by ETBF colonization. b) Immunofluorescence confocal images and c) quantitative analysis of E‐cadherin density in *B. fragilis* colonized µGut. d) Immunofluorescence confocal images and e) quantitative analysis of β‐catenin nuclear translocation in *B. fragilis* colonized µGut. f) Magnified view (100x) of µGut epithelial cells surrounded by *B. fragilis* biofilm showing intense β‐catenin localization around ETBF9. g) A schematic of ETBF‐triggered pStat3 nuclear translocation. h) Immunofluorescence confocal imaging of pStat3 nuclear translocation and i) quantitative analysis of nuclear pStat3 density in *B. fragilis* colonized µGut. j) 100x view of NTBF and ETBF9 biofilm enclosed µGut epithelial cells displaying specific pSTAT3 translocation by ETBF9. k) A schematic of ETBF‐induced NF‐κB activation and nuclear translocation. l) NF‐κB nuclear translocation and m) nuclear NF‐κB density analysis in *B. fragilis* colonized µGuts imaged by immunofluorescence confocal microscopy. n) High‐magnification image (100x) of NF‐κB localization in µGut epithelial cells wrapped around NTBF and ETBF9. o) Immunofluorescence staining of γH2AX in µGut epithelium and p) quantitative analysis of cellular γH2AX density in *B. fragilis* colonized µGuts. All scale bars represent 10 µm. P‐value indicate *p < 0.05; **p < 0.01; ***p < 0.001, NS→ *p* > 0.05.

E‐cadherin cleavage and its subsequent degradation has been attributed to the binding of BFT to colonic epithelial cells in ETBF‐colonized mice and BFT‐treated cells^[^ ^49^ ^]^ (Table S2, Supporting Information). In our study, defined expression of E‐cadherin at the cell‐cell junctions was detected in bacteria‐free control and NTBF colonized µGut (**Figure** **4**a,b). However, a spotty and diffused distribution of E‐cadherin was observed in both ETBF8‐ and ETBF9 colonized µGuts, indicating that ETBF colonization contributes to E‐cadherin cleavage in µGut. The quantitative analysis highlighted that E‐cadherin density was significantly (*p* < 0.01 for ETBF8 and *p* < 0.001 for ETBF9) reduced for both enterotoxigenic strains (Figure 4c). The ETBF9 colonized µGut showcased the lowest E‐cadherin density, suggesting a strong correlation with high levels of active BFT produced by ETBF9 (Figure 3j).

E‐cadherin cleavage reportedly induces the release and translocation of β‐catenin to the nucleus of colonic epithelial cells^[^ ^50^, ^54^ ^]^ (Figure 4a and Table S2, Supporting Information). The ETBF9 colonized µGut showcased a notable increase in the nuclear translocation of β‐catenin (P‐value < 0.05), in contrast to the control, NTBF, and ETBF8 models (Figure 4d,e). Considering that the degree of E‐cadherin cleavage is closely correlated to the level of β‐catenin translocation, enhanced β‐catenin in the ETBF9 model is attributed to the highest level of E‐cadherin cleavage as shown in Figure 4c. High‐magnification imaging also revealed that the cells surrounded by ETBF9 exhibited highly accumulated β‐catenin in the cell nucleus whereas NTBF‐enclosed µGut epithelial cells did not display β‐catenin translocation (Figure 4f).

ETBF‐induced pSTAT3 nuclear translocation is another pre‐oncogenic signaling event reported only in vivo models^[^ ^51^, ^55^ ^]^ (Figure 4g and Table S2, Supporting Information). In the µGut, nuclear pSTAT3 translocation levels were significantly higher (≈130 fold increase, *p* < 0.001) for ETBF9 colonization in comparison to the control and other strains (Figure 4h,i). Visualization of the µGut cells using high‐magnification confocal imaging showed significant pStat3 translocation signals in the µGut epithelial cells surrounded by ETBF9, whereas no pStat3 localization was observed for those surrounded by NTBF (Figure 4j).

NF‐κB signaling pathway has been shown to be activated by ETBF colonization in vivo^[^ ^51b^ ^]^ or by BFT stimulation in epithelial cells^[^ ^51^, ^52^ ^]^ (Figure 4k and Table S2, Supporting Information). Additionally, E‐cadherin cleavage by BFT can further trigger MAPKs‐initiated NF‐κB pathway, contributing to the enhanced nuclear translocation of NF‐κB.^[^ ^52^, ^56^ ^]^ However, other factors such as a hypoxic condition^[^ ^57^ ^]^ or the presence of bacteria^[^ ^57^, ^58^ ^]^ can also initiate NF‐κB signaling, which needs to be distinguished from ETBF‐induced NF‐κB activation. In fact, we observed enhanced levels of nuclear translocation levels of NF‐κB p65 in the bacteria‐free control and NTBF colonized µGut, attributed to hypoxic stress and the general presence of bacteria (*p* < 0.005) (Figure 4l,m; Figure S8, Supporting Information). However, ETBF colonization, specifically ETBF9, induced significantly enhanced NF‐κB p65 translocation in µGut, strongly suggesting that ETBF9 and high levels of BFT produced by ETBF9 contributed to a significant level of p65 translocation (ETBF8 *p* < 0.01, ETBF9 *p* < 0.001). An enlarged view of the model also showcased the ETBF9 encircling µGut epithelial cells with pronounced NF‐κB accumulation in the cell nucleus, while the presence of NTBF did not induce detectable levels of NF‐κB (Figure 4n).

Finally, we evaluated ETBF‐induced DNA damage via γH2AX immunofluorescence. ETBF‐induced DNA damage has previously been reported in animal models^[^ ^41b^ ^]^ and BFT‐stimulated cells^[^ ^59^ ^]^ (Figure 4k and Table S2, Supporting Information). The µGut colonized by ETBF9 displayed significant amounts of γH2AX‐positive epithelial cells (≈12 fold as compared to the control, *p* < 0.001), whereas NTBF and ETBF8 colonized µGuts displayed a lower fold increase, indicating DNA damage affected by high levels of BFT (Figure 4o,p).

In conclusion, we successfully demonstrated and visualized several pathogenic signaling pathways activated by live ETBFs in the 3D µGut within our GMoC. Compared to NTBF, ETBF9 had the most pronounced effect on activating pathogenic signaling pathways in the µGut. Given that BFT toxin is primarily responsible for ETBF‐induced pathogenic signaling events, our results highlight that the GMoC is a reliable platform for simulating ETBF‐induced gut pathogenesis and its mechanisms by utilizing live bacteria and it's in situ‐produced virulence factors.

### 2.5 Inter‐Microbial Competition Diminishes ETBF Colonization and Pathogenesis in µGut, Preserving the Healthy State of µGut

In a healthy human intestine, commensal gut microbiota can resist the colonization of potentially harmful microorganisms,^[^ ^60^ ^]^ which lowers the risk of intestinal infection, and inflammation, preventing disease development.^[^ ^61^ ^]^ One of the key mechanisms for colonization resistance is inter‐microbial competition, where different bacterial species compete for limited resources such as nutrients and physical space for growth in the gut.^[^ ^62^ ^]^ This competition between different bacterial species helps to prevent harmful bacteria from overgrowing and disrupting balanced gut microbiota in the gut.^[^ ^60b^ ^]^

**Figure 5:**
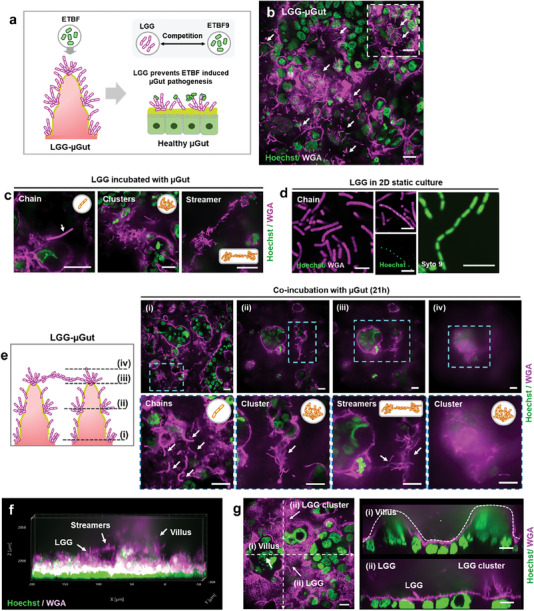
Establishment of the dynamic probiotic‐enriched µGut (LGG‐µGut), showcasing the formation of distinctive LGG structures. a) A schematic illustrating pre‐treatment of the µGut with beneficial LGG (LGG‐ µGut) resisting ETBF colonization and growth by inter‐microbial competition, preserving the healthy state of µGut. b) Top view of the LGG‐µGut showing the abundance of LGG (Pink strands). The high background from the epithelial cells is due to the gram stain, WGA, staining the mucin on the epithelial cells. The inset figure shows protruding LGG strands from the mucin layer on the µGut epithelium. **c)** Different LGG structures formed in the LGG‐µGut. d) Chain forming LGG in MRS agar media (Scale bar, 5 µm). e) Confocal Z‐stack images of the LGG‐µGut from the bottom i) to top planes iv) of the crypt‐villus axis showcasing various LGG structures. f) 3D visualization of the LGG‐ µGut showing the abundant presence of LGG covering the villus‐crypt axis. g) Cross‐section images of the LGG‐ µGut showcasing i) undisrupted villi‐like structure covered with ii) LGG chains and clusters. All scale bars represent 20 µm unless otherwise indicated.

To simulate colonization resistance of commensal bacteria against harmful microbes and explore inter‐microbial compeititon between them,   we enriched µGut with beneficial microbes *Lactobacillus rhamnosus* GG (LGG) to estalish the LGG‐enriched μGut. Subsequently, pathogenic ETBF was introduced to the μGut and we observed the interaction between LGG with ETBF, along with their influence on µGut health (**Figure** **5**a). To begin, we established LGG‐enriched µGut (LGG‐µGut) by introducing LGG into the µGut and incubating it for 21 h to allow the full growth of LGG on the μGut surface. Fluorescent gram stains were used to visualize LGG‐µGut, which showed the formation of various LGG phenotypic structures such as single‐strand chains, thick clusters of LGG resembling LGG biofilm,^[^ ^63^ ^]^ and interconnected thread‐like structures that are similar to bacterial streamers induced by flow^[^ ^64^ ^]^ and geometry^[^ ^65^ ^]^ (Figure 5b,c). These various LGG phenotypic structures contrast the simple LGG chains observed in 2D culture plates^[^ ^66^ ^]^ (Figure 5d). Through Z‐stack analysis, we observed that LGG colonized on the µGut in various forms, covering from the crypt to the villus. (Figure 5e,f, and Video S7, Supporting Information). In addition, LGG clusters protruding from a villus formed a thread‐like streamer with other LGG clusters, demonstrating the formation of complex networks by probiotic bacteria in the μGut (Figure 5e,f, and Video S7, Supporting Information). Notably, despite abundant LGG growth, the 3D architecture of the µGut remained intact without indications of disruption observed in ETBF‐colonized μGut , highlighting the protective effect of probiotic bacteria on the intestine^[^ ^67^ ^]^ and the successful establishment of viable LGG‐enriched µGut (Figure 5g and Video S7, Supporting Information).

**Figure 6:**
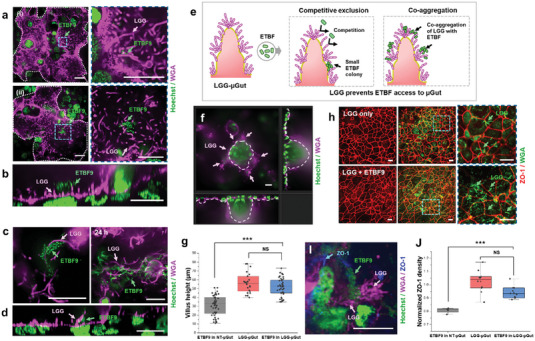
LGG‐µGut exhibit resistance against ETBF colonization through effective competitive mechanisms, protecting the µGut from ETBF‐induced disruption. a) Diminished ETBF9 colonization in the LGG‐µGut evidenced by the presence ofi) only a few ETBF9 and ii) small‐sized ETBF9 colonies on the LGG‐colonized surface. b) A cross‐section image of an ETBF9 colony in the LGG‐µGut showing the attachment of ETBF9 on LGG. c) Top view and d) cross‐section of ETBF9 co‐aggregating with LGG chains or LGG biofilms. e) A schematic diagram of the two direct colonization resistance mechanisms of the LGG‐µGut against ETBF. i) LGG inhibits ETBF adhesion or proliferation by competitive exclusion or (ii) LGG co‐aggregate with ETBF9, preventing ETBF9 access to the µGut surface. f,g) Cross‐section and height measurement of villi‐like structures in the LGG‐µGut after ETBF9 colonization. h,i) Immunofluorescence confocal images of ZO‐1 and j) normalized ZO‐1 density in the LGG‐µGut after ETBF9 colonization. All scale bars represent 20 µm unless otherwise indicated.

On the established LGG‐µGut, we introduced ETBF9, the most virulent ETBF strain and observed the interaction between LGG and ETBF, along with their collective impact on μGut. Only small numbers or small‐sized colonies of ETBF9 were found in the LGG‐µGut, indicating that pre‐colonized LGG actively inhibits the attachment and proliferation of ETBF9 in the LGG‐µGut (**Figure** **6**a). Cross‐section images revealed that inhibition mechanisms of LGG against ETBF9, as LGG prevent direct binding of ETBF9 to the µGut by attaching to ETBF9, indicating intermicrobial competition for the µGut surface (Figure 6b).

Additionally, we observed that various phenotypic structures of LGG co‐aggregated with ETBF9, further hindering its access to the µGut (Figure 6c,d). In contrast to the abundant colonization of ETBF9 and the formation of large‐sized colonies on the untreated µGut (NT‐µGut) (Figure 3b,c,f), our results highlight that the resistance mechanisms of LGG‐µGut significantly diminished ETBF colonization. This can be attributed to competitive exclusion, where LGG competes for the binding surface (Figure 6a,b), or co‐aggregation, where LGG forms co‐aggregate with ETBF^[^ ^60b^ ^]^ (Figure 6c–e). The presence of LGG provided effective protection, as evidenced by minimal disruption of the 3D architecture (Figure 6f,g) and barrier integrity of the LGG‐µGut in response to ETBF9 (Figure 6h‐j) in contrast to the significant damages observed in the µGut without the presence of LGG (Figure 6f and Figure 3). As a result, the LGG‐µGut exhibited taller villi (≈50 µm) compared to shortened villi (≈30 µm) observed in NT‐µGut (Figure 6f,g). Furthermore, ETBF9 did not induce ZO‐1 redistribution in the LGG‐µGut, suggesting improved barrier integrity (Figure 6h–j).

In summary, our study showcased the colonization resistance of a beneficial gut microbe in the µGut against pathogen colonization, effectively preventing pathogen‐induced μGut disruption. These findings highlight the utility of the GMoC  in accessing the effectiveness of microbe‐based therapeutics, unveiling underlying mechanisms, and elucidating kety microbial interactions, providing valuable insights for designing targeted and effective microbe‐based therapies.   .

## 3 Discussion

The significance of gut microbiome in human disease and health has garnered increasing attention in recent years. While various gut models have been developed to mimic gut physiology and pathogenesis, there has been a notable gap in the availability of in vitro co‐culture models capable of visualizing and dissecting the causative and preventive behaviors of live gut microbes and microbial communities, along with their underlying mechanisms that influence gut health.

Herein, we have developed a GMoC that leverages high‐magnification imaging to explore the intricate interactions between gut and gut microbes. The simple and non‐sophisticated design of the parallelized GMoC facilitates easy fabrication and good reproducibility across chips with the potential for high‐throughput scalability. Direct contact between collagen and Caco‐2 cells has been reported to improve cell polarization and differentiation.^[^ ^68^ ^]^ Such architecture led to altered microenvironmental cues for cells located farther away from the collagen gel, leading to differential cell behavior in various sections of the chip. This , in turn, impacted the reproducibility and physiological relevance of the epithelium. Through empirical observations, we determined that minimal contact between collagen gel and Caco‐2 cells favored cell proliferation and long‐term culture without creating a non‐uniform cell microenvironment. Consequently, consistent Caco‐2 cell polarization, differentiation, and 3D organization were observed across the entire gut chip.

The µGut, generated from Caco‐2 cells, self‐organizes into a physiologically relevant 3D differentiated epithelium, providing a consistent and reproducible 3D gut microbial habitat for gut microbiome studies. The great imaging capability of the GMoC allows us to visualize the various bacterial behaviors, encompassing attachment, growth exhibiting various phenotypic structures, and the development of pathogenicand beneficial behaviors. Furthermore, we were able to monitor changes in the 3D μGut in response to various bacteria, observing the μGut disruption such as villus shortening, Zo‐1 redistribution, and pathogenic signaling induced by pathogenic bacteria. Conversely, the μGut maintained a healthy state in the presence of beneficial bacteria.   ,  t S High‐magnification imaging allows us to visualize inter‐microbial interactions among different gut microbial species, wherein probiotic bacteria protect the gut by exerting colonization resistance against pathogenic speceis through detailed mechanisms such as competitive exclusion and co‐aggregation. These intricate mechanisms were not previously understood with current gut‐on‐a‐chip technology

Our GMoC empowers us to gain deeper insights into the unknown roles of individual microbial species and microbial communities within the gut microbiota, shedding light on their impact on overall health at a mechanistic level. Through this platform, we can investigate inter‐microbial interactions and spatial organization of gut microbial communities in a 3D context, while closely monitoring their behavioral changes in response to environmental stimuli under a controlled environment. The incorporation of multiple microchannels also serves as a versatile multi‐well platform, enabling us to pool experimental replicates, thus increasing RNA or protein yields for downstream analysis, which is often challenging in most microfluidic systems.

Recreating the intricate complexity of the gut microbiota in vitro has proven to be a challenging endeavor. In vitro studies often face limitations in capturing the full microbial diversity present in the human intestine due to the existence of unculturable microbial species in vitro. Nonetheless, substantial efforts have been dedicated to constructing synthetic gut microbiota or microbial community comprising 10–100 species of bacteria at the strain level, aiming to reconstitute stable synthetic gut microbiota using data‐driven models and germ‐free mice models.^[^ ^69^ ^]^ In a noteworthy prior study, researchers employed a synthetic bacterial community comprising 14 species of cultivable gut bacteria and observed responses to dietary changes. Interestingly, only four species of bacteria displayed changes in abundance, influencing mucus degradation and pathogen susceptibility.^[^ ^70^ ^]^ These findings emphasize the importance of investigating a pair‐wise of higher‐order interactions between gut microbial communities, presenting new opportunities to develop targeted interventions and strategies for modulating gut microbiota.

To establish a standardized platform with consistent and reproducible gut‐microbe interfaces for high‐throughput assays, we opted to use Caco‐2 cells to mimic the structural and functional characteristics of the intestines.^[^ ^71^ ^]^ While primary human‐derived intestinal epithelial cells offer greater physiological relevance, their application as a standardized platform for gut microbiome studies is constrained by low reproducibility and variability, and a time‐consuming incubation process. In contrast, a cell line‐based system presents an alternative biomimetic gut model, offering significant advantages such as uniformity, reproducibility, and efficient experimentation turnover. Despite originating from colorectal adenocarcinoma, Caco‐2 cells have demonstrated the ability to undergo gene expressions change upon polarization, resulting in a closer alignment with the characteristics observed in normal colon tissue.^[^ ^72^ ^]^ Moreover, previous studies demonstrated that 3D structured Caco‐2 cells, cultured under physiodynamic conditions, exhibit a highly differentiated intestinal epithelial phenotype similar to normal epithelial phenotypes through reprogramming.^[^ ^19^, ^73^ ^]^ Our results support these findings as we observed the presence of multiple types of differentiated cells, which are not typically reported in Caco‐2 cells. Enhanced imaging further allows us to reveal the clear spatial patterning of these differentiated cells. Combining this evidence with our data, it becomes evident that the Caco‐2 cells offers significant advantages as in vitro micro physiological system, overcoming some of the limitations of conventional cell‐line‐based systems that can only recapitulate a specialized function of specific cell types.

While previous studies have indicated that fluid shear stimulation of the Caco‐2 cells through apical and basal fluid flow is crucial for inducing 3D morphogenesis of Caco‐2 or its derived clone C2BBe1^[^ ^19^, ^22^, ^74^ ^]^ by regulating the concentrations of the Wnt antagonist Dickkopf‐1 (DKK‐1) and expression of the Wnt receptor, frizzled‐9 (FZD9),^[^ ^22b^ ^]^ our studies revealed that shear stress induction on the apical surface alone is sufficient to facilitate the 3D structuring and differentiation of the Caco‐2. These findings suggests the existence of additional underlying mechanisms influencing Caco‐2 morphogenesis. Furthermore, our observations indicate that apical shear flow can modulate the genetic and protein expression levels of MUC2, indicating that fluid shear serves as an important mechanical factor in regulating MUC2 synthesis and altering the characteristics of Caco‐2 cells, aligning with previous evidence of gene expression changes in response to mechanical cues.^[^ ^68^, ^73^ ^]^

Studying gut microbiota in 3D culture substrate is critical for obtaining a realistic understanding of the spatial organization and interactions among various microbes and host cells within their spatial context. Our study, leveraging this 3D gut model, unveiled previously unseen behaviors of LGG, including the formation of biofilms and complex networks, which were not observed in 2D cultures. Given that biofilm formation of probiotics is considered beneficial for promoting long‐term colonization and persistence,^[^ ^75^ ^]^ this distinct LGG behavior observed in the 3D dynamic model system may be linked to their protective role against pathogens in the human intestine. Furthermore, our cell‐based 3D µGut model allowed us to differentiate varying levels of host response induced by strain‐level pathogenicity of ETBFs, and NTBF, a capability not achievable with 2D gut models or other non‐cell‐based 3D scaffolds for microbial culture.

The selection of ETBF as our model system was driven by its strong association with gut inflammation and colorectal cancer.^[^ ^36^, ^41^ ^]^ Our Gut microbiome‐on‐a‐chip enables us to culture *B. fragilis* in a 3D µGut for up to 24 h under hypoxic conditions, enabling the observation of early carcinogenic signaling events induced by ETBF. In our study, we demonstrated previously unseen in vivo‐like behavior of live *B. fragilis*, including production of a BFT toxin in vitro, resulting in µGut disruption and activation of tumorigenic signaling pathways using live bacteria.^[^ ^36b^ ^]^ The ability to replicate the pathogen's behavior and gut response using live microbes in vitro setting overcomes the challenges of conventional approaches that require prior identification and purification when investigating the unknown roles of gut microbes. It is worth noting that, compared to purified BFT toxin, ETBF took longer to initiate gut pathogenesis in the µGut model, possibly due to the time required for ETBF to grow and produce the BFT in the GMoC (Table S2, Supporting Information).

Generating oxygen gradients on the GMoC remains a challenge, especially for long‐term studies of gut‐microbe cultures, where the human gut epithelium and bacteria may require different oxygen levels. In our study, to avoid hypoxia‐induced signals from prolonged incubation, we focused on capturing only early signaling events activated by ETBF. However, the development of the on‐chip oxygen gradient generation holds promise for engineering a physiologically relevant oxygen environment, accommodating multiple microbial species with varying oxygen requirements. The implementation of such a system could offer comprehensive and long‐term studies of gut‐microbe interactions, providing valuable insights into the complex dynamics of gut microbiota within a realistic oxygen environment.

Despite our primary aim of developing the standardized in vitro gut model, our µGut structure, consisting of the crypt‐villus units, mimics the key structures of the small intestine, which differ from the structure of the large intestine. In this proof of principle study, we utilized ETBF and LGG to demonstrate microbe‐induced gut pathogenesis and microbial intervention. However, it is important to note that different gut microbes reside in different parts of the intestine, and the geometry of small and large intestines may influence bacterial behaviors due to the differing environment from their natural habitat. Therefore, to study the correct behavior of gut microbes and gut microbial communities, it is essential to consider the structure and environment of the intestine, resembling its natural habitat.

While our study focused on inter‐bacterial competition as a form of direct colonization resistance, it is important to acknowledge that gut microbiota is composed of a diverse range of microbial species with various types of interactions between them. These interactions can have remarkable impacts on determining the function of the gut microbiota, ultimately resulting in a healthy state of the gut.^[^ ^76^ ^]^ Exploring these largely unexplored relationships can yield valuable insights into effective gut microbiota modulation and the development of therapeutic intervention.^[^ ^77^ ^]^

In summary, we have successfully developed a GMoC with great imaging capability and scalability, enabling in‐depth causal and mechanistic studies between a gut microbial community and the gut. Our reproducible and functional 3D µGut model closely resembles the 3D architecture of the intestine, featuring spatially patterned differentiated functional cells, providing an efficient and effective biomimetic scaffold for culturing gut microbes and investigating their impact on the gut. By culturing the carcinogenic microbe, ETBF, in the GMoC, we were able to induce in vivo‐like gut pathogenesis, eliciting various gut responses ranging from morphological changes to the activation of carcinogenic signaling.

Notably, pre‐treating the µGut with beneficial bacteria LGG effectively protected it from ETBF through competitive mechanisms, preserving its healthy state by diminishing the colonization and growth of the pathogenic ETBF. This discovery underscores the potential of our GMoC to unravel complex relationships among different microbial species within gut microbiota and study their collective impact on the human intestine. Such knowledge will deepen our understanding of microbe‐related disease and facilitate the development of new therapeutic interventions for gut microbiota modulation. We believe that our GMoC can serve as a valuable model system to explore unknown roles and relationships of gut microbial species, contributing to filling the gaps in our understanding of the gut microbiota.

## 4 Experimental Section

### Fabrication of the GMoC

The GMoC comprised 15 unique channels that were arranged in a 3 × 5 format. PDMS soft lithography was performed with a SU‐8 photoresist‐based Si mold that was fabricated by photolithography. Briefly, a positive relief of the channel design was fabricated using SU‐8 based photolithography on a silicon (Si) wafer following which the Si mold was silanized with trichloro(1H,1H,2H,2H‐perfluorooctyl) silane (Sigma–Aldrich) to protect any damages to the SU‐8 design during the repeated PDMS chip preparation. PDMS was prepared using the elastomer and curing agent (10:1 by weight) and poured over the SU‐8 mold, degassed, and cured at 70 °C for 2 h. The PDMS was carefully released from the Si wafer and inlets and outlets were defined using biopsy punches. To complete the chip preparation, the PDMS channels were bonded to a glass coverslip by oxygen plasma treatment (Tergeo, Pie Scientific LLC). While array design signifies parallelization and high‐throughput processing, the individual µGut channels were spaced such that the PDMS can be cut in‐between, and bonded and operated as unique individual chips enabling operational flexibility if downsizing the number of parallelized channels was needed.

### Microfluidic Cell Culture

The Caco‐2 cell line was a kind gift from Professor Gigi N. C. Chiu from the Department of Pharmacy at the National University of Singapore. The Caco‐2 cell was cultured in EMEM (Lonza) supplemented with 20% FBS (Lonza) and 1% penicillin/streptomycin (Lonza). The Caco‐2 cells between passages 61 and 70 were used for all experiments. Before on‐chip cell culture, the chip was sterilized under UV light for 30 min. The collagen gel matrix (rat tail Type I collagen (Ibidi GmbH) was prepared following the manufacturer's protocol. Briefly, 5 mg mL^−1^ collagen matrix was titrated using 1 M NaOH and upon achieving a neutral pH, the mixture was loaded into the collagen channel of the chip and promoted gelation by incubating at 37 °C for 30 min. The microchannel was coated with 50 µg mL^−1^ of rat tail Type I collagen (Ibidi GmbH) for 1.5–2 h and subsequently washed with the serum‐free media. The resuspended Caco‐2 cells were seeded into the coated channel at a concentration of 8 × 10^6^ cells mL^−1^ and allowed to be attached overnight. The media was perfused continuously from the next day at 45 µl h^−1^ (shear stress ≈0.034 dyne cm^−2^) for 6–7 days to form the 3D µGut, using an external syringe pump (Chemyx pumps, USA). For the static control, the culture media was replenished every day for the same growth period as the perfused culture.

### Bacteria culture

The *E. coli* with GFP tag (ATCC 25922^TM^) was purchased from ATCC. The *E. coli* was cultured in LB media containing 100 µl mL^−1^ Ampicillin as recommended by the ATCC handling procedure. The two enterotoxigenic *Bacteroides fragilis* (ETBF), ATCC 43858 (2‐078382‐3) and ATCC 43859 (20799‐3) were purchased from ATCC. The non‐enterotoxigenic *B. fragilis* (NTBF, ATCC 25285) was a kind gift from Professor Kevin SW Tan from the Department of Microbiology and Immunology at the National University of Singapore. All *B. fragilis* were cultured in Trypticase soy media (Merck) supplemented with defibrinated sheep blood (Thermo Fisher Scientific, R54020) under an anaerobic condition (AnaeroGen, Oxoid) at 37 °C. *Lactobacillus rhamnosus* GG (LGG, ATCC 53103) was purchased from ATCC and cultured in MRS agar (Merck) under 5% CO_2_ at 37 °C.

### Co‐Culture of µGut and Bacteria

For µGut‐bacteria co‐culture, the cell culture media was replaced on Day 5 with the antibiotic‐free culture media (EMEM with 20% FBS) containing 75 µg mL^−1^ of porcine mucin (Sigma–Aldrich M2378). Perfusion culture was resumed for at least 12 h to ensure that no antibiotic traces were left within the channels before bacteria inoculation. For bacteria inoculation, the cultured bacterial cells were harvested and adjusted to the optimized density (1‐2 × 10^7^ cfu mL^−1^) by resuspending them to the antibiotic‐free cell culture medium (EMEM with 20% FBS). The resuspended bacteria are added into the disconnected microchannel and incubated without flow for 1.5–3 h to promote bacterial attachment. The microchannel was washed with media twice and connected to the syringe pump (Chemyx pumps, USA) to allow constant flow of antibiotic‐free culture medium. The co‐culture in the GMoC was incubated under normoxic or 1% hypoxic conditions depending on the oxygen requirements of the bacteria. For LGG‐enriched µGut, the density‐adjusted LGG (10^8^ cfu mL^−1^) was inoculated into a disconnected chip and incubated for 5 h without flow followed by resuming perfusion for 21 h. Resuspended ETBF (1‐2 × 10^7^ cfu mL^−1^) in antibiotic‐free media was added into the established LGG‐enriched µGut within the disconnected culture channel and incubated for 3 h to promote attachment under 1% hypoxic condition. After washing the channel, the GMoC was connected to the syringe pump and further incubated under 1% hypoxic condition for 24 h.

### Immunofluorescence Staining

For immunofluorescence studies, the µGut culture in the GMoC was fixed with 4% PFA (Pierce) for 10 mins and permeabilized with 0.2% Triton‐X100 (Sigma–Aldrich) for 15 mins. The µGut culture was blocked with 1% BSA (Sigma–Aldrich) for 2 h and washed twice with the same blocking buffer. Subsequently, the microfluidic culture was incubated with a primary antibody dissolved in the blocking buffer at 4 °C overnight. After washing the culture with the blocking buffer twice, a secondary antibody in the blocking buffer was added into the chip and further incubated at room temperature (r.t.) for 1 h in a dark environment. The nucleus and actin filaments of the Caco‐2 and μGut culture were stained with Hoescht 33 342 (Thermo Fisher Scientific, 62 249) and Phalloidin‐iFluor 647 (Abcam, ab176759), respectively. Primary antibodies for different targets were purchased from various manufacturers; Anti‐ Vilin1[R814] (Cell Signaling Technology, 2369), Anti‐ZO‐1 (Invitrogen, 61–7300), Anti‐Sucrase Isomaltase [A‐12] (Santa Cruz Biotechnology, sc‐393424), Anti‐MUC2 [996/1] (Abcam, ab11197), Anti‐Chromogranin A (Abcam, ab1560), Recombinant Anti‐Lysozyme, [EPR2994(2)] (Abcam, ab108508), Anti‐SOX9 [D8G8H] (Cell Signaling Technology, 82 630), Anti‐Ki67 [8d5] (Cell Signaling Technology, 9449), Anti‐ Phospho‐Stat3 (Tyr705) [D3A7] XP® (Cell Signaling Technology, 9145), Anti‐ E‐Cadherin [24E10] (Cell Signaling Technology, 3195), Anti‐β‐Catenin [D10A8] (Cell Signaling Technology, 8480), Anti‐NF‐κB p65 [D14E12], (Cell Signaling Technology, 8242), Anti‐Phospho‐Histone H2A.X [Ser139] (20E3), (Cell Signaling Technology, 9718), Anti‐HIF‐1α [D1S7W], (Cell Signaling Technology, 36 169). The secondary antibodies were purchased as follows; Goat Anti‐Mouse IgG H&L (Alexa Fluor® 488) (Abcam, ab150113), Goat Anti‐Rabbit IgG H&L (Alexa Fluor® 488) (Abcam, ab150077) depending on reactivity. To assess the viability of µGut in hypoxic conditions, the culture was stained with Live/Dead™ Viability/Cytotoxicity Kit, for mammalian cells (Invitrogen™, L3224) followed by visualization with spinning disk confocal microscopy. The viability of bacteria was also evaluated with Live/Dead™ Baclight ™ Bacterial Viability Kit (Invitrogen™, L7012) with imaging analysis. At least 3 GMoC were used in each experiment and the images were taken at no fewer than 5 different sites within the cell culture chamber for further quantification. All experiments were repeated twice for validation.

### Fluorescent Gram Staining

A live Bacterial gram stain kit (Biotium, 32000) was used to stain the co‐culture of µGut with bacteria by modifying the manufacturer's staining protocol. Briefly, the disconnected microchannel of the µGut‐bacteria culture was washed twice with BSA‐NaCl buffer (0.25% BSA, 0.15 M NaCl in PBS), followed by incubation with CF™594 WGA for 10 min under the dark. After removing excess dye by washing with BSA‐NaCl buffer, DAPI was added to the microchannel and further incubated for 5 min, followed by 4% PFA fixation.

### Morphological Analysis

Different microscopes were utilized based on the study. Bright‐field images of the µGut architecture and time‐course images of Caco‐2 culture were recorded using an inverted phase‐contrast microscope (Nikon Eclipses T*i* ; Nikon with Evolve^TM^ camera). The morphology of µGut structure and microvilli were imaged by a spinning disk confocal microscopy comprising an inverted microscope (Ti‐E, Nikon), a spinning disk scan head (CSU‐W1; Yokogawa), an sCMOS camera (Prime95B; Teledyne Photometrics) and a laser system (iLaunch; GATACA Systems). The µGut samples were visualized with CFI plan Apo objective under laser excitation 404/561/642 nm and scanned with Z‐series at step size 1 µm. The images were processed with MetaMorph (Molecular Devices), IMARIS (Bitplane Scientific software), and Image J. 3D mapping of the Caco‐2 cells and bacteria as well as cell and villi height measurements were performed using IMARIS. All image‐based quantification for cell signaling pathways were performed using an ImageJ script developed in‐house. For cell signaling quantification, all the samples were normalized against the hypoxic control, except for E‐cadherin, which was plotted without normalization.

### Aminopeptidase Assay

Aminopeptidase activity of the µGut was determined using L‐alanine‐4‐ nitroanilide hydrochloride (A4N)^[^ ^78^ ^]^ (Sigma–Aldrich A9325). After 6 days of µGut formation, the microbiome was disconnected from the pump and further incubated with 1.5 mM A4N solution in a culture medium for 1 h at 37 °C under the static condition. After removing the solution from the microchannel, the presence of the cleaved product, 4‐nitroaniline, was measured at 405 nm using a NanoDrop spectrophotometer (Thermo Fisher Scientific). To quantify average enzymatic activity, at least three chips were used for all measurements.

### Detection of Mucin

Production of mucin by µGut in the perfused microfluidic chip was evaluated by the Alcian blue staining method.^[^ ^22^, ^79^ ^]^ Briefly, µGut culture in the device under static or perfused conditions was fixed with 4% PFA(w/v). The Alcian blue 8GX (pH 2.5) (Sigma–Aldrich, 66 011) solution was diluted with 3% Acetic acid (Sigma–Aldrich) and introduced over PFA fixed cells in the culture chamber at 30 µl h^−1^ for 16 h followed by washing with 1×PBS. The stained culture was imaged using an inverted microscope (Olympus IX73) mounted with an EMCCD camera (Andor iXon X3). The light absorbance by the stained cell layer was determined using Image J by normalizing against a blank chip (without cells) to quantify the mucin secreted by the cells. For quantification of Alcian blue, at least 3 individual chips were imaged, and each experiment is repeated at least twice. To visualize the mucin layer of µGut‐ bacteria co‐culture, Wheat Germ Agglutinin Alexa Fluor 555™ conjugate (Invitrogen™, W32464) was used as described in previous studies.^[^ ^18^, ^80^ ^]^ Briefly, the culture in the µGut chip was washed with Hank's balanced salt solution (HBSS) (Lonza), and 5 µg mL^−1^ of WGA solution was added into the channel and further incubated for 10 min at 37 °C. The culture was fixed with 4% PFA (Sigma–Aldrich), stained with 1x Hoescht 33 342 (Thermo Fisher Scientific) for 15 min at r.t., and visualized using the spinning disk confocal microscope.

### Gene Expression Analysis

After disconnecting the chip from the pump, the µGut was washed with PBS twice and total RNA was extracted using RNeasy Plus Micro Kit (Qiagen, 74 034). RNA concentration and purity were determined using a Nanodrop spectrophotometer (Thermo Fisher Scientific). Subsequently, the resulting RNA was converted to cDNA via iScript™ Advanced cDNA Synthesis Kit (Bio‐Rad, 1 725 038) with the Verti® 96 well Thermal cycler (Applied Biosystem). RT‐qPCR amplification of the gene was performed using SsoAdvanced™ Universal SYBR® Green Supermix (Bio‐Rad, 1 725 271) on CFX96 Touch Real‐Time PCR Detection System (Bio‐Rad) following the manufacturer's recommended protocol (30 s at 95 °C followed by 40 cycles of 30 s at 95 °C and 15 s at 60 °C). The PCR primers for the MUC2 gene were purchased from a commercial source (Origene, HP206138) and the primers for the bft gene^[^ ^81^ ^]^; FW 5′‐AAG GGC TGG ATG GCT TTA CT‐3′, REV 5′ GGG ATA CAT CAG CTG GGT TG‐3′, 16S FW 5′‐CAG TCT‐TGA GTA CAG TAG AGG TGG‐3′ 16S REV: 5′ GTG GAC TAC CAG GGT ATC TAA TCC‐3′) were obtained from Integrated DNA Technologies (Integrated DNA Technologies, Singapore). The qPCR results were analyzed using Bio‐Rad CFX Manager software (Bio‐Rad). The PCR‐amplified product of the bft gene from all *B. fragilis* strains was analyzed in 2% agarose gel electrophoresis by staining with SYBR™ safe DNA gel stain (Invitrogen S33102) and visualized with iBright FL1000 imaging system (Invitrogen).

### Western Blotting of BFT

The co‐culture of the µGut with *B. fragilis* was harvested using Allprep® DNA/RNA/Protein Mini kit (Qiagen, 80004) following the manufacturer's protocol. The resulting protein mixture was separated by SDS‐PAGE using 4%–20% Mini‐Protean TGX gels (Bio‐Rad, 4561094) and subsequently transferred to 0.2 µm PVDF membrane (Bio‐Rad, 1704156) using Trans‐Blot Turbo system (Bio‐Rad). The membrane was blocked with 5% BSA (w/v) and incubated with *B. fragilis* Fragilysin Polyclonal Antibody (Invitrogen™, PA5‐117596) overnight at 4 °C. The membrane was subsequently incubated with Anti‐Rabbit IgG, HRP‐linked Antibody (Cell Signaling Technology, 7074) for 1 h at r.t. and detected with Clarity Max Western ECL substrate (Bio‐Rad, 170 562) using iBright FL1000 imaging system (Invitrogen). As a loading control, β‐Actin was used (Cell Signaling Technology, 4967) with Anti‐mouse IgG, HRP‐linked Antibody (Cell Signaling Technology, 7076).

### Statistical Analysis

The data in this study were generated from a minimum of 5 repetitive chips (*n* = 5) and all numerical data were expressed as mean ± standard deviation (SD) unless explicitly stated otherwise. The statistical significance between the two groups were evaluated using a Student's t‐test, with a significance level of *p* < 0.05 indicating a significant difference.

## Conflict of Interest

The authors declare no conflict of interest.

## Supporting information

Supporting Information

Supplemental Video 1

Supplemental Video 2

Supplemental Video 3

Supplemental Video 4

Supplemental Video 5

Supplemental Video 6

Supplemental Video 7

Supplemental Video 8

## Data Availability

Research data are not shared.
