# Supplementary material for: Dissecting Gut‐Microbial Community Interactions using a Gut Microbiome‐on‐a‐Chip
Source: Adv Sci (Weinh). 2024 Feb 27;11(20):2302113. doi: 10.1002/advs.202302113 (PMC11132043; doi:10.1002/advs.202302113)
Supplement: Supplementary file 1 — Supporting Information [file ADVS-11-2302113-s004.pdf]

ADVANCED SCIENCE
Open Access

Supporting Information

for *Adv. Sci.*, DOI 10.1002/advs.202302113

Dissecting Gut-Microbial Community Interactions using a Gut Microbiome-on-a-Chip

*Jeeyeon Lee, Nishanth Menon and Chwee Teck Lim**

Dissecting Gut-Microbial Community Interactions using a Gut Microbiome-on-a-Chip

Jeeyeon Lee[1¶], Nishanth Menon[2¶], Chwee Teck Lim[1,2,3]*

[1] Institute for Health Innovation and Technology (iHealthtech), National University of Singapore, Singapore 117599, Singapore

[2] Department of Biomedical Engineering, National University of Singapore, Singapore 117583, Singapore

[3] Mechanobiology Institute, National University of Singapore, Singapore 117411, Singapore

*Corresponding author

E-mail: ctlim@nus.edu.sg

[¶] These authors contributed equally.

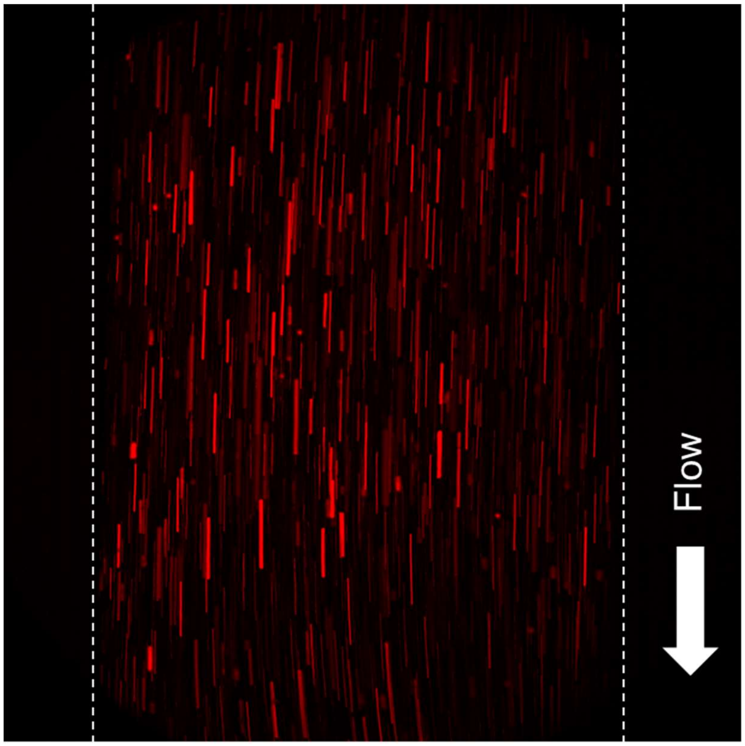

Figure S1. Max intensity projection of 1 μm fluorescent beads perfusion at 45 μl/h.

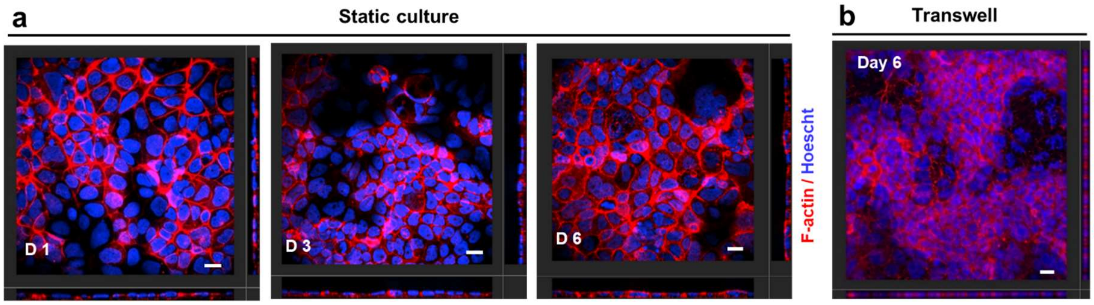

Figure S2. Caco-2 monolayer formation under the static condition.
a) Time-lapse images of Caco-2 monolayer formation in static μGut chips imaged by brightfield and confocal microscopy. b) The thin monolayer of Caco-2 formed in static Transwell® culture (Scale bar, 20 μm).

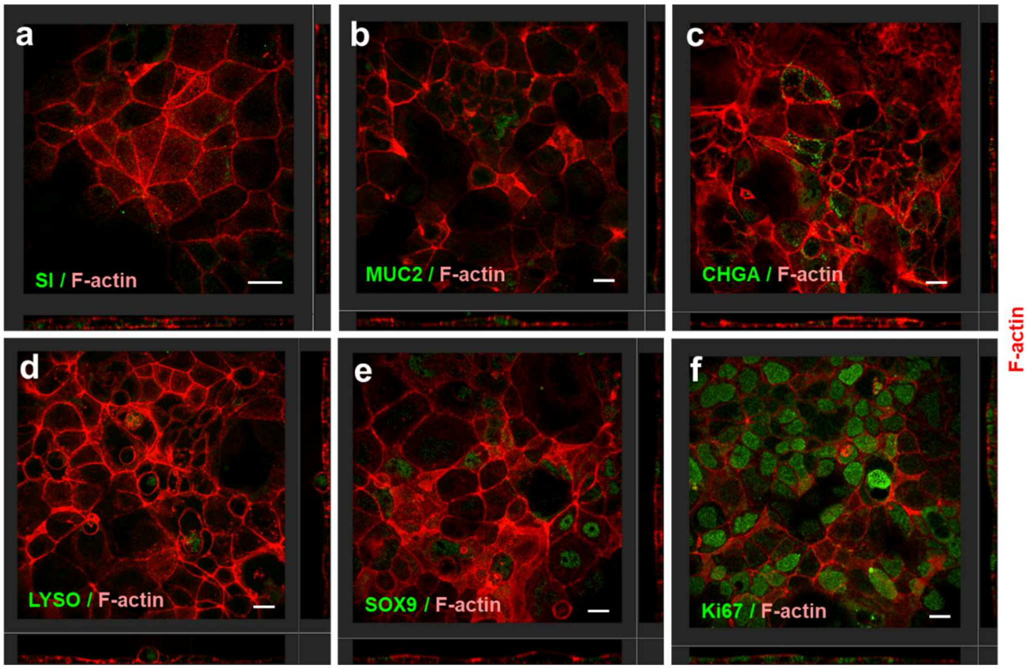

Figure S3. Immunofluorescence confocal images of key intestinal markers in Caco-2 monolayers cultured in static μGut chips. a) enterocytes (SI), b) goblet cells (MUC2), c) chromogranin A (CHGA), d) lysozyme (LYSO), e) stem cells (SOX9), f) proliferating cells (Ki67) (Scale bar, 20 μm).

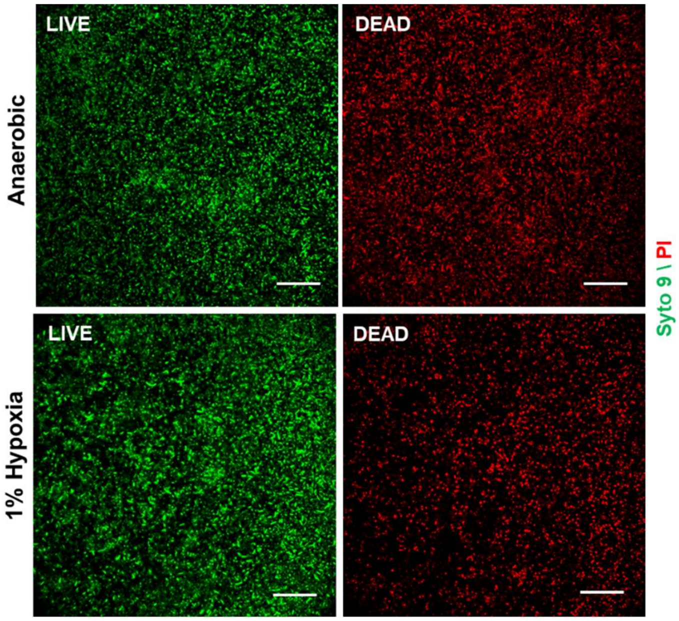

Figure S4. Viability of *B. fragilis* (NTBF) after 24 h incubation under anaerobic and 1% hypoxic conditions (Scale bar, 200 μm).

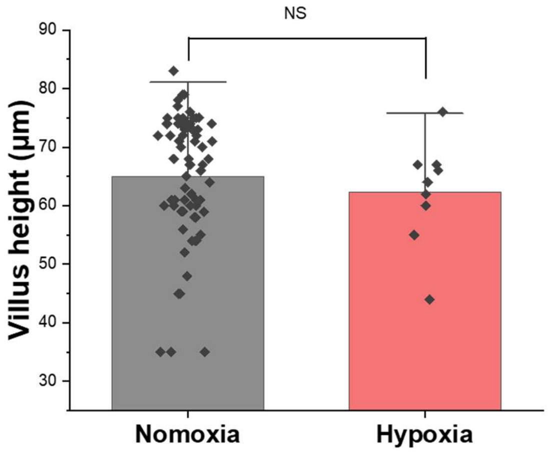

Figure S5. Comparison of villus heights of the μGuts after 24 h incubation under normoxic and 1% hypoxic conditions.

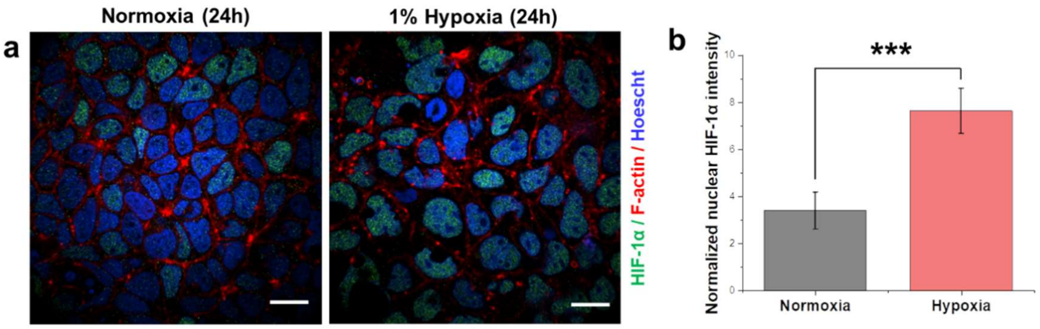

Figure S6. Comparison of HIF-1α expression in the μGut after 24 h incubation under normoxia and 1% hypoxia. a) Immunofluorescence confocal images of HIF-1α expression in the μGut and b) normalized HIF-1α densities (***$p < 0.001$) (Scale bar, 20 μm).

Table S1. In various model systems, ETBF and BFT-induced morphological changes of the intestinal epithelial cells.

**Type of changes**	***In vivo* model**	**BFT-treated cells**
**Morphology**	Extensive detachment and rounding of surface epithelial cells, as well as shortened villi in ETBF-colonized and BFT-treated animal tissue[1]	Altered cell morphology (cell rounding, loss of cell height, apical drift of cell nuclei) in monolayers of T84 cell or HT29/C1 cell[2]
**ZO-1 redistribution**	No reported studies	ZO-1 redistribution in BFT treated HT29/C1 cell line[3]

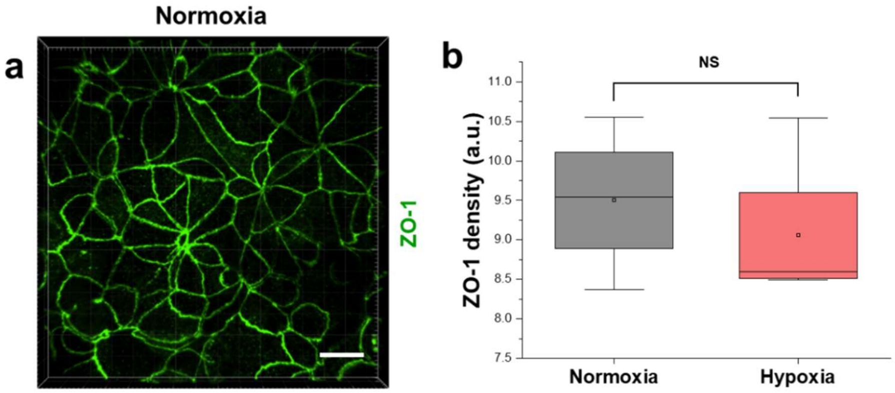

Figure S7. ZO-1 redistribution of μGuts cultured under normoxia and 1% hypoxia. a) An immunofluorescence confocal microscopy image of ZO-1 redistribution in μGut cultured under normoxic conditions (NS→ p>0.05). b) Comparison of ZO-1 densities of μGuts cultured under different oxygen conditions.

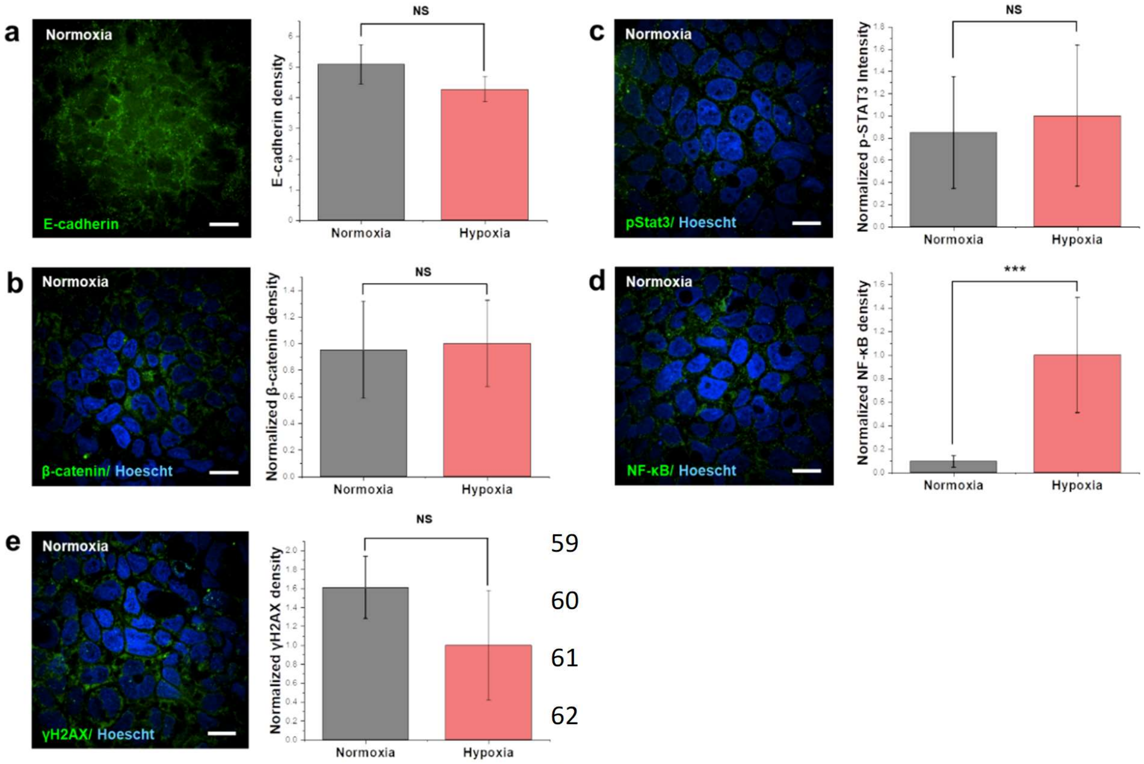

Figure S8. Immunofluorescence confocal images and quantitative analysis of various ETBF-induced signaling molecules in μGuts after 24 h incubation under normoxia. The density of signaling molecules was compared to that under 1% hypoxia. a) E-cadherin b) β-Catenin c) p-STAT3 d) NF-κB e) γH2AX (***p < 0.001, NS→ p>0.05) (Scale bar, 20 μm).

Table S2. ETBF-indued carcinogenic signaling pathways reported in *in vivo* models and BFT toxin-treated cells.

Signaling pathway	In vivo model	BFT toxin-treated cell models
**E-cadherin cleavage**	18~ 24 h, after inoculation in ETBF-colonized mice[4]	1 h after BFT treated HT29/C1 cell line[5]
**β-catenin nuclear translocation**	No reports of *in vivo* models	a. 3~6 h after BFT treatment in HT29/C1 cells[6]<br><br>b. 12 h in HCT116 and CCD841CoN cells[7]
**Pstat3 nuclear translocation**	16~24 h after inoculation in ETBF colonized wild-type C57BL/6 and Min mice[8]	a. No detection *in vitro* model<br><br>b. No detection in BFT-treated HT29/C1 cells up to 72 h and polarized T84 cells within 24 h[8b]
**NF-κB p65 nuclear translocation**	Day 7 of post-infection in ETBF colonized C57BL/6 mice[8a]	6 h after BFT stimulation in HT29/C1 cells[8a, 9]
**DNA damage**	Day 4 of inoculation in ETBF colonized AOM mice[10]	Within 6 h after BFT treatment in T84 cells[11]

Movie S1: Visualizing perfusion in the μGut channel using 1μm beads.

Movie S2: Caco-2 μGut growth under static and perfusion conditions.

Movie S3: Formation of 3D villus-like structure in μGut.

Movie S4: NTBF adhesion and proliferation in μGut.

Movie S5: Adhesion and proliferation of ETBF8 in μGut.

Movie S6: Adhesion and proliferation of ETBF9 in μGut.

Movie S7: ETBF biofilm formation in the μGut.

Movie S8: *Lactobacillus rhamnosus* GG (LGG) enriched μGut (LGG- μGut).

Reference

[1] a) C. L. Sears, L. L. Myers, A. Lazenby, R. L. Van Tassell, *Clin. Infect. Dis.* **1995**, *20 Suppl 2*, S142; b) R. J. Obiso, Jr., D. M. Lyerly, R. L. Van Tassell, T. D. Wilkins, *Infect. Immun.* **1995**, *63* (10), 3820.

[2] F. G. Chambers, S. S. Koshy, R. F. Saidi, D. P. Clark, R. D. Moore, C. L. Sears, *Infect. Immun.* **1997**, *65* (9), 3561.

[3] S. Wu, K. C. Lim, J. Huang, R. F. Saidi, C. L. Sears, *Proc. Natl. Acad. Sci. U. S. A.* **1998**, *95* (25), 14979.

[4] K. J. Rhee, S. Wu, X. Wu, D. L. Huso, B. Karim, A. A. Franco, S. Rabizadeh, J. E. Golub, L. E. Mathews, J. Shin, R. B. Sartor, D. Golenbock, A. R. Hamad, C. M. Gan, F. Housseau, C. L. Sears, *Infect. Immun.* **2009**, *77* (4), 1708.

[5] S. Wu, K. J. Rhee, M. Zhang, A. Franco, C. L. Sears, *J. Cell. Sci.* **2007**, *120*, 1944.

[6] S. Wu, P. J. Morin, D. Maouyo, C. L. Sears, *Gastroenterology* **2003**, *124* (2), 392.

[7] J. I. Jeon, S. H. Ko, J. M. Kim, V. J. Torres, *Infect. Immun.* **2019**, *87* (11).

[8] a) L. Chung, E. T. Orberg, A. L. Geis, J. L. Chan, K. Fu, C. E. DeStefano Shields, C. M. Dejea, P. Fathi, J. Chen, B. B. Finard, A. J. Tam, F. McAllister, H. Fan, X. Wu, S. Ganguly, A. Lebid, P. Metz, S. W. Van Meerbeke, D. L. Huso, E. C. Wick, D. M. Pardoll, F. Wan, S. Wu, C. L. Sears, F. Housseau, *Cell Host Microbe* **2018**, *23* (3), 421; b) E. C. Wick, S. Rabizadeh, E. Albesiano, X. Wu, S. Wu, J. Chan, K. J. Rhee, G. Ortega, D. L. Huso, D. Pardoll, F. Housseau, C. L. Sears, *Inflamm. Bowel. Dis.* **2014**, *20* (5), 821; c) S. Wu, K. J. Rhee, E. Albesiano, S. Rabizadeh, X. Wu, H. R. Yen, D. L. Huso, F. L. Brancati, E. Wick, F. McAllister, F. Housseau, D. M. Pardoll, C. L. Sears, *Nat. Med.* **2009**, *15* (9), 1016.

[9] J. M. Kim, S. J. Cho, Y. K. Oh, H. Y. Jung, Y. J. Kim, N. Kim, *Clin. Exp. Immunol.* **2002**, *130* (1), 59.

[10] C. M. Dejea, P. Fathi, J. M. Craig, A. Boleij, R. Taddese, A. L. Geis, X. Wu, C. E. DeStefano Shields, E. M. Hechenbleikner, D. L. Huso, R. A. Anders, F. M. Giardiello, E. C. Wick, H. Wang, S. Wu, D. M. Pardoll, F. Housseau, C. L. Sears, *Science* **2018**, *359* (6375), 592.

[11] A. C. Goodwin, C. E. D. Shields, S. Wu, D. L. Huso, X. Wu, T. R. Murray-Stewart, A. Hacker-Prietz, S. Rabizadeh, P. M. Woster, C. L. Sears, R. A. Casero, *Proc. Natl. Acad. Sci. U. S. A.* **2011**, *108* (37), 15354.
